# Structural Requirements in the Hemagglutinin Cleavage Site-Coding RNA Region for the Generation of Highly Pathogenic Avian Influenza Virus

**DOI:** 10.3390/pathogens10121597

**Published:** 2021-12-09

**Authors:** Yurie Kida, Kosuke Okuya, Takeshi Saito, Junya Yamagishi, Aiko Ohnuma, Takanari Hattori, Hiroko Miyamoto, Rashid Manzoor, Reiko Yoshida, Naganori Nao, Masahiro Kajihara, Tokiko Watanabe, Ayato Takada

**Affiliations:** 1 Division of Global Epidemiology, International Institute for Zoonosis Control, Hokkaido University, Sapporo 001-0020, Japan; yurie-kida@czc.hokudai.ac.jp (Y.K.); kokuya@vet.kagoshima-u.ac.jp (K.O.); t.saito@czc.hokudai.ac.jp (T.S.); hattoritakanari@czc.hokudai.ac.jp (T.H.); hirom@czc.hokudai.ac.jp (H.M.); manzoor@czc.hokudai.ac.jp (R.M.); ryoshida@czc.hokudai.ac.jp (R.Y.); kajihara@czc.hokudai.ac.jp (M.K.); 2 Division of Collaboration and Education, International Institute for Zoonosis Control, Hokkaido University, Sapporo 001-0020, Japan; junya@czc.hokudai.ac.jp; 3 Technical Office, International Institute for Zoonosis Control, Hokkaido University, Sapporo 001-0020, Japan; aikoh@czc.hokudai.ac.jp; 4 Division of International Research Promotion, International Institute for Zoonosis Control, Hokkaido University, Sapporo 001-0020, Japan; n-nao@czc.hokudai.ac.jp; 5 One Health Research Center, Hokkaido University, Sapporo 060-0818, Japan; 6 Department of Molecular Virology, Research Institute for Microbial Diseases, Osaka University, Osaka 565-0871, Japan; tokikow@biken.osaka-u.ac.jp; 7 International Collaboration Unit, International Institute for Zoonosis Control, Hokkaido University, Sapporo 001-0020, Japan

**Keywords:** influenza virus, highly pathogenic avian influenza virus, hemagglutinin, cleavage site, RNA secondary structure, nucleotide insertion

## Abstract

Highly pathogenic avian influenza viruses (HPAIVs) with H5 and H7 hemagglutinin (HA) subtypes are derived from their low pathogenic counterparts following the acquisition of multiple basic amino acids in their HA cleavage site. It has been suggested that consecutive adenine residues and a stem-loop structure in the viral RNA region that encodes the cleavage site are essential for the acquisition of the polybasic cleavage site. By using a reporter assay to detect non-templated nucleotide insertions, we found that insertions more frequently occurred in the RNA region (29 nucleotide-length) encoding the cleavage site of an H5 HA gene that was predicted to have a stem-loop structure containing consecutive adenines than in a mutated corresponding RNA region that had a disrupted loop structure with fewer adenines. In virus particles generated by using reverse genetics, nucleotide insertions that created additional codons for basic amino acids were found in the RNA region encoding the cleavage site of an H5 HA gene but not in the mutated RNA region. We confirmed the presence of virus clones with the ability to replicate without trypsin in a plaque assay and to cause lethal infection in chicks. These results demonstrate that the stem-loop structure containing consecutive adenines in HA genes is a key molecular determinant for the emergence of H5 HPAIVs.

## 1. Introduction

Influenza A viruses (IAVs) belong to the genus *Alphainfluenzavirus* in the family Orthomyxoviridae. The IAV genome consists of eight segments (PB2, PB1, PA, NP, HA, NA, M, and NS) of negative-sense, single-stranded RNA. IAVs are divided into subtypes based on the antigenicity of their two surface glycoproteins, hemagglutinin (HA) and neuraminidase (NA). Currently, 16 HA (H1–H16) and nine NA (N1–N9) subtypes are found in wild aquatic birds [1,2]. Non-pathogenic avian influenza viruses (AIVs) with the H5 or H7 HA subtype, which are maintained in the wild waterfowl reservoir, are occasionally transmitted to chickens through terrestrial birds and acquire high pathogenicity to chickens via multiple infections and circulation in domestic poultry [3,4].

Infection with highly pathogenic avian influenza viruses (HPAIVs) causes high mortality rates in poultry and tremendous economic losses to the poultry industry around the world. The H5N1 HPAIV that was first found in Hong Kong in 1997 has been causing outbreaks in poultry since around 2003, mainly in Southeast Asia and the Middle East, and has spread to Europe and Africa [5,6,7]. The H5N1 HPAIV infects not only birds but also various mammals, including humans, with high mortality rates [8]. The first reported human case of H5N1 virus infection occurred in 1997 [9]. Since its reemergence in 2003, H5N1 HPAIV has been transmitted to humans sporadically and 862 human cases of H5N1 HPAIV infection, including 455 deaths, have been reported (as of 22 July in 2021, http://www.who.int/, accessed on 24 July 2021) [10].

HA is a virulence determinant of AIVs. The HA gene is translated as the precursor HA0, which is subsequently cleaved into the HA1 and HA2 subunits by host proteases [11]. HA cleavage is prerequisite for the virus to be infectious [12]. Low pathogenic avian influenza viruses (LPAIVs) possess a monobasic amino acid motif in their HA cleavage site, which is recognized by trypsin-like proteases localized in some mucosal tissues. Therefore, the replication of LPAIVs is restricted to the respiratory and intestinal tracts where these enzymes are present. In contrast, HPAIVs contain multiple basic amino acids at the HA cleavage site [13]. This polybasic cleavage motif is cleaved by ubiquitous proteases such as furin and PC6 [14,15]. Hence, HPAIVs can replicate systemically and cause severe disease in gallinaceous poultry.

The HPAIVs that have been naturally isolated to date are restricted to either the H5 or H7 subtype, with a few exceptions; an H10 virus that lacks the polybasic cleavage site but shows high pathogenicity in an intravenous pathogenicity index test and an H4 virus that has a cleavage site with 3 arginines but shows low virulence in chickens [16,17]. The conversion of LPAIVs of the H5 and H7 subtypes to the highly pathogenic phenotype occurs when basic amino acid residues are introduced at the HA cleavage site through substitution or insertion of nucleotides that create a polybasic cleavage motif. Interestingly, artificial introduction of multiple basic amino acids into the HA cleavage site of an H6 LPAIV strain allows the virus to replicate in the absence of trypsin in vitro and to cause systemic infection of chickens [18]. An H9 virus also exhibits high virulence for chickens after introduction of a pair of dibasic amino acid residues into the HA cleavage site and subsequent passages in chickens [19]. Some AIV strains of other HA subtypes (H2, H4, H8, and H14) similarly show highly pathogenic phenotypes in an appropriate genetic background upon the artificial introduction of multiple basic amino acids at the HA cleavage site [20]. These studies indicate that the structural capacity of the HA molecule to have a polybasic cleavage motif is shared among many HA subtypes, but only H5 and H7 viruses appear to be genetically predisposed to accepting nucleotide insertions to create codons for multiple basic amino acid residues in their HA cleavage sites.

In the 1990s, involvement of the secondary structure of the viral RNA region that encodes the HA cleavage site was suggested as a mechanism underlying the emergence of HPAIVs [21,22]. It has been proposed that the acquisition of multiple basic amino acids is due to polymerase slippage on template regions with stable secondary structures [23,24,25], or simple substitution [26]. An alternative proposal, reported for the H7 subtype, is recombination of the HA RNA genome with other RNA of host or virus origin [27,28,29,30,31]. Although these studies hypothesized that RNA secondary structure and polymerase errors are involved in the generation of multiple basic amino acids at the cleavage site, empiric evidence was mostly absent. We have shown that the RNA region around the cleavage site of most low-pathogenic H5 and H7 viruses isolated from waterfowl contains characteristic stem-loop structures including more than eight adenine and/or guanine nucleotides. A recent study revealed that a particular lineage of the H7 viruses shared the conserved stem-loop structure, suggesting a possible role of the RNA structure in polymerase errors [32]. The loop sequence consisting of consecutive adenines/guanines may be favorable to create codons for lysine and/or arginine residues (e.g., AAA, AAG, AGA, and AGG) around the HA cleavage site [33].

**Figure 1:**
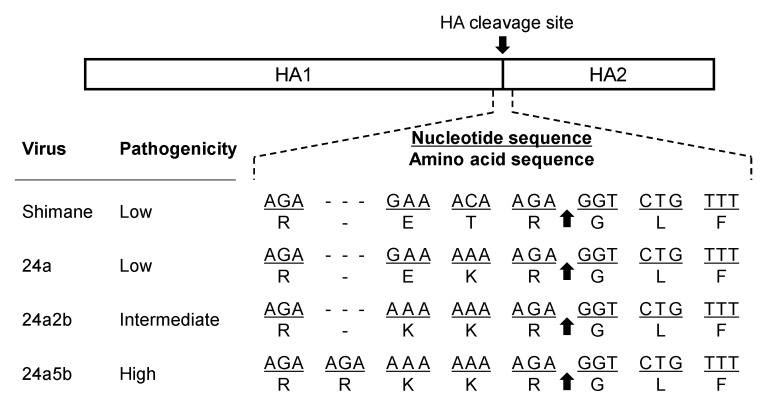
HA cleavage site sequences of the Shimane strain and its variants obtained during passaging in chickens. The pathogenicity determined by intranasal infection of chickens with each virus is shown [34]. The arrow indicates the cleavage site between HA1 and HA2. Nucleotide sequences (mRNA-sense orientation) at positions 1043 to 1063 (Shimane, 24a, and 24a2b) and 1043 to 1066 (24a5b) and the corresponding amino acid sequences are shown. Dashes are included to adjust the sequence alignment.

The H5 virus A/whistling swan/Shimane/499/1983 (H5N3) (Shimane) was originally isolated as an LPAIV and became highly pathogenic through serial passages in chickens [34]. The amino acid sequence at the HA cleavage site of Shimane was of the typical low pathogenic type (RETR/G), but after passage in chickens, the virus gradually acquired basic amino acid residues (i.e., 24a, 24a2b, and 24a5b viruses had REKR/G, RKKR/G, and RRKKR/G, respectively) at the cleavage site and became an HPAIV that was 100% fatal in infected chickens (Figure 1). It was experimentally demonstrated that adenine insertions into the viral RNA positions forming the stem-loop structure frequently occurred during the replication of the virus and that the number of nucleotides in a loop and the number of consecutive adenines in the predicted stem-loop structure affected the frequency of nucleotide insertions [33]. In the present study, we found that the stem-loop structure containing a consecutive adenine sequence was essential for the insertion of multiple nucleotides that create additional codons in the viral RNA sequence corresponding to the HA cleavage site, leading to the acquisition of basic amino acid residues at that site. Virus clones with a highly pathogenic phenotype were generated through insertion of multiple nucleotides into the loop region. These data, together with our previous study [33], provide direct evidence that the presence of the stem-loop structure in the viral RNA region encoding the HA cleavage site is a key molecular determinant for the emergence of HPAIVs with the H5 subtype in nature.

## 2. Results

### 2.1. Association between Nucleotide Insertions and Predicted Stem-Loop Structures Containing Adenine Stretches in HA Genes

**Figure 2:**
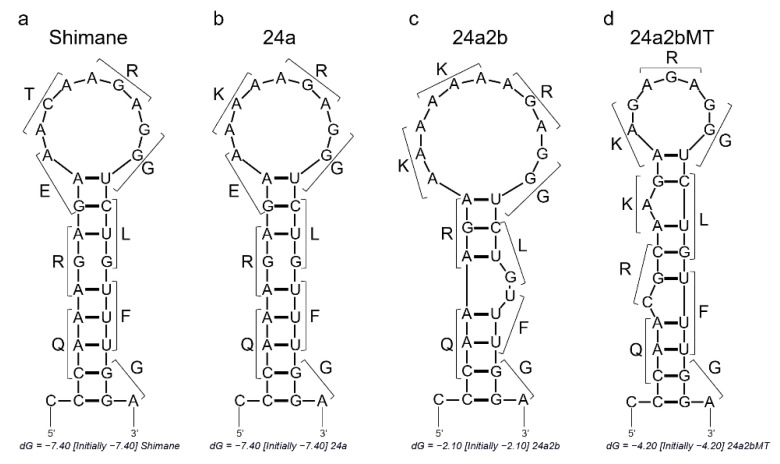
Predicted RNA structures of the cleavage site sequences of HA genes. RNA secondary structures were predicted by using mfold from the UNAFold Web Server. The structures of the RNA regions (mRNA-sense orientation) around the HA cleavage sites of Shimane (**a**), 24a (**b**), 24a2b (**c**), and 24a2bMT (**d**) and the amino acids corresponding to each codon are shown.

As mentioned, the Shimane strain gradually acquired basic amino acid residues at its HA cleavage site and became an HPAIV (24a5b) via 24a and 24a2b viruses. The RNA sequences encoding the HA cleavage sites of Shimane, 24a, and 24a2b have three, six, and eight adenine runs, respectively (Figure 1). The 24a and 24a2b viruses acquired these additional adenines due to nucleotide substitutions during passaging in chickens [34]. We have previously shown that the RNA sequences encoding the cleavage site of Shimane and its variants are expected to form stem-loop structures, and that 24a2b, which has eight consecutive adenine residues, has an expanded loop structure (Figure 2) and a higher frequency of nucleotide insertions into adenine runs than Shimane and 24a [33]. To further clarify the significance of the stem-loop structure and adenine runs of 24a2b for accelerated nucleotide insertion, we compared the efficiency of nucleotide insertion among RNA regions with different stretches of adenines and different sizes of the loop structures.

**Figure 3:**
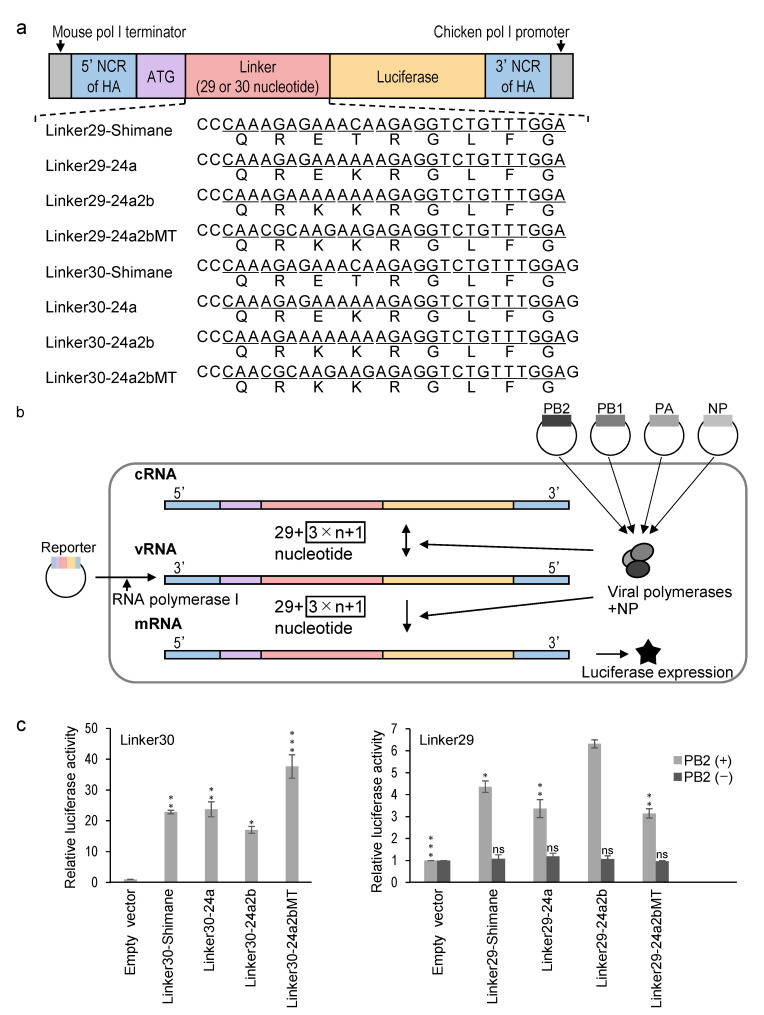
Nucleotide insertions into RNA sequences around the HA cleavage site detected in the reporter assay. (**a**) The reporter plasmids contained the chicken RNA polymerase I promoter, mouse RNA polymerase I terminator, A/Puerto Rico/8/34 (H1N1) (PR8) HA segment-derived non-coding region (NCR), and the firefly luciferase gene. Linkers (29 or 30 nucleotides) derived from the RNA sequences encoding amino acids around the HA cleavage sites of Shimane, 24a, 24a2b, and 24a2bMT were inserted between the start codon and the firefly luciferase gene lacking the start codon. (**b**) QT6 cells were transfected with each reporter plasmid, the thymidine kinase promoter-Renilla luciferase transfection control reporter plasmid (pRL-TK), and expression plasmids encoding PB2, PB1, PA, and NP (or empty plasmid instead of PB2 expression plasmid). (**c**) Luciferase activities relative to empty vector are shown. Results are expressed as the mean ± standard error of the firefly luciferase activity, normalized by the Renilla luciferase activity in three independent experiments. Statistical significance compared to empty vector (Linker30), empty vector (Linker29 PB2 minus), or Linker29-24a2b (Linker29 PB2 plus) was calculated using one-way analysis of variance (ANOVA) followed by Dunnett’s test (* *p* < 0.05, ** *p* < 0.01, *** *p* < 0.0001) (ns, nonsignificant).

To evaluate the association between nucleotide insertions and stem-loop structures containing consecutive adenines, we used a previously established reporter assay to detect non-templated nucleotide insertions [33] (Figure 3). In this system, a negative-sense vRNA template is transcribed from the reporter plasmid by RNA polymerase I, and mRNA and cRNA are produced by the expressed polymerases and NP. In the reporter plasmid, the firefly luciferase gene lacking its start codon is inserted downstream of the 29 or 30-polynucleotide linker region corresponding to the sequence of the RNA regions encoding the HA cleavage site (Figure 3a). Since the firefly luciferase gene following the 29-linker sequence is out of frame, luciferase is expressed only when a single (or 3 × *n* + 1) nucleotide is inserted into the linker region of the mRNA, cRNA, and/or vRNA to form an open reading frame (ORF) (Figure 3b). We constructed reporter plasmids containing linker regions for Shimane (Linker29/30-Shimane), 24a (Linker29/30-24a), and 24a2b (Linker29/30-24a2b). Reporter plasmids with linker regions that had the mutated sequence of 24a2b were also constructed (Linker29/30-24a2bMT). The Linker29/30-24a2bMT plasmid had fewer consecutive adenine residues and a smaller predicted loop structure, but had the same amino acid sequence as 24a2b (Figure 2c,d and Figure 3a).

We first found high luciferase expression in the QT6 cells transfected with Linker30 plasmids. We then confirmed that the luciferase activity of the cells transfected with the Linker29-24a2b plasmid was significantly higher than that of the cells transfected with the Linker29-Shimane or Linker29-24a plasmid. As expected, significantly lower luciferase expression was observed in the cells transfected with the Linker29-24a2bMT plasmid than in those transfected with the Linker29-24a2b plasmid (Figure 3c).

### 2.2. Insertions of Codons for the Creation of Polybasic Cleavage Sites in 24a2b HA

We then investigated whether nucleotide insertions into the stem-loop region of the 24a2b HA gene would result in the addition of codons in-frame for basic amino acid residues to create polybasic cleavage sites. For this purpose, infectious virus particles possessing the Shimane, 24a2b, or 24a2bMT HA gene (rgPR8/Shimane, rgPR8/24a2b, and rgPR8/24a2bMT, respectively) were generated and propagated in MDCK cells, and their RNA genomes were analyzed by deep sequencing. We found that adenines were inserted into the consecutive adenine sequences in the RNA regions encoding the cleavage sites of rgPR8/Shimane and rgPR8/24a2bMT (0.0042% and 0.0000% in 701,562 and 4,010,838 reads, respectively). Nucleotide insertions into the eight consecutive adenine sequences in this RNA region of rgPR8/24a2b were observed at a much higher frequency (4.83% in 4,248,638 reads) than those in the Shimane HA gene. Of the 4,248,638 reads that contained the nucleotide sequence of the RNA region encoding the HA cleavage site, 3,982,290 reads (93.731%) corresponded to the original sequence (QRKKR/GLF) of 24a2b, 41,412 reads (0.975%) had point mutations without nucleotide insertion, 212,768 reads (5.008%) had single or double nucleotide insertions causing frameshifts, and 12,168 reads (0.286%) had nucleotide insertions that generated in-frame additional codons in the region. Among the 12,168 reads, there were insertions of multiple nucleotides into the RNA region to produce additional codons that could create various HA cleavage sites, including some with multiple basic amino acid residues (e.g., QRKKKR/GLF, QRKKRKKR/GLF, and QRRKKR/GLF) (Table 1). Nucleotide insertions associated with addition of basic amino acid codons were not observed in the genome of infectious virus particles possessing the 24a2bMT HA gene (rgPR8/24a2bMT).

### 2.3. Insertion of Basic Amino Acid Residues at the HA Cleavage Site during the Replication of rg24a2b in Cultured Cells

To further investigate the effect of the loop size and the number of consecutive adenines on the creation of additional basic amino acids at the HA cleavage site, we generated recombinant 24a2b (rg24a2b) and its mutant virus (rg24a2bMT) that had fewer consecutive adenines and a reduced loop size but had the same amino acid sequence as 24a2b (Figure 2d). These viruses were produced by transfection of 293T cells with the plasmids and were propagated once in MDCK cells in the presence of trypsin. The viral titers in the supernatants were 3.2 × 10^8^ PFU/mL (rg24a2b) and 8.3 × 10^7^ PFU/mL (rg24a2bMT), indicating that both viruses replicated well in MDCK cells in the presence of trypsin.

**Figure 4:**
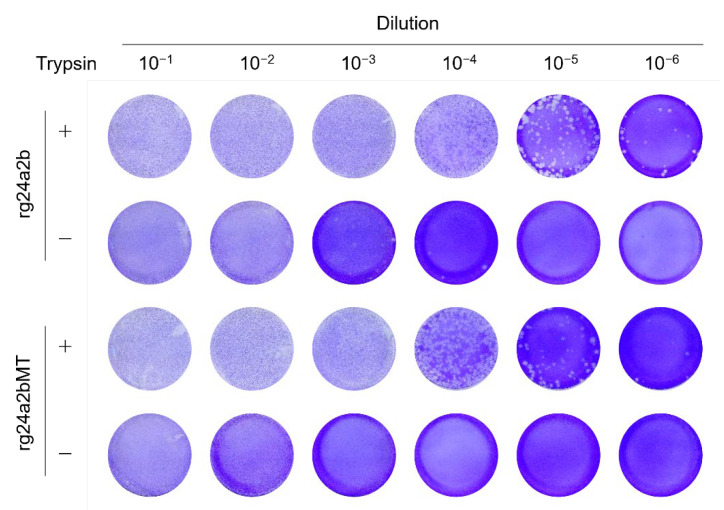
Plaque-forming abilities of the viruses in the presence and absence of trypsin. MDCK cells were infected with serial dilutions (100 μL each) of 24a2b and 24a2bMT in the presence (+) and absence (−) of trypsin (5 μg/mL). Results are representative of at least two independent experiments.

Interestingly, rg24a2b formed plaques at 10^−3^ and 10^−4^ dilutions of the virus stock even in the absence of trypsin, whereas no visible plaques were observed in rg24a2bMT-infected cells without trypsin in the same condition (Figure 4), as was the case with rgShimane and rg24a (data not shown). At the dilutions of 10^−1^ and 10^−2^, visible plaques could not be clearly observed in the absence of trypsin both for rg24a2b and rg24a2bMT since substantial amounts of the cells were dead due to single-step infection with the inoculum virus. RNA was extracted from nine virus clones that formed plaques in the absence of trypsin, and the amino acid sequences of their HA cleavage sites were determined. As expected, we found insertions of three amino acids (KKR, KRK, or RKK) or a single amino acid (R) into the HA cleavage site of the 24a2b virus (PQRKKR/GLF), which created polybasic cleavage sites PQRKKRKKR/GLF (eight of the nine clones) and PQRRKKR/GLF (one of the nine clones) (Table 2). These data demonstrated that replication of rg24a2b, but not rg24a2bMT, in transfected 293T cells or during subsequent passage in MDCK cells resulted in the insertion of basic amino acid residues at the HA cleavage site, allowing a small population of the virus to replicate in cultured cells in the absence of trypsin.

### 2.4. Selection of Viruses That Acquired Additional Basic Amino Acid Residues at the HA Cleavage Site in Chicks

**Figure 5:**
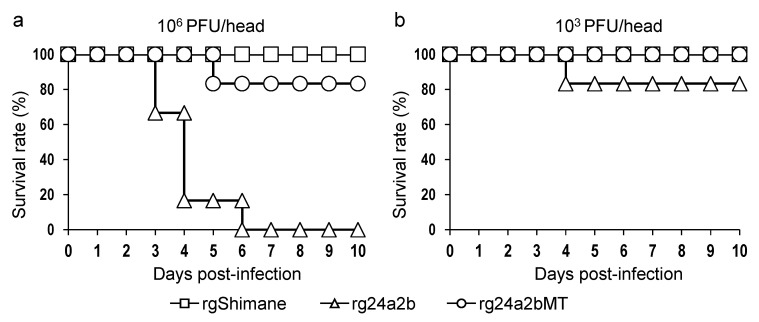
Survival rates of chicks intracerebrally inoculated with rgShimane, rg24a2b, or rg24a2bMT. The chicks were intracerebrally inoculated with 100 µL of PBS containing 10^6^ (**a**) or 10^3^ (**b**) PFU of each virus and observed for 10 days after inoculation.

Finally, we inoculated rg24a2b and rg24a2bMT into chicks to confirm the presence or absence of viruses with a highly pathogenic phenotype. When chicks were infected intracerebrally with 10^6^ PFU/head of the virus, rg24a2b killed all of the infected chicks within 6 days of infection, whereas five of six chicks inoculated with rg24a2bMT and all of the rgShimane-infected chicks survived (Figure 5a). In contrast, the survival rate of chicks infected with 10^3^ PFU/head of rgShimane or rg24a2bMT was 100%, whereas one of the rg24a2b-infected chicks died 4 days after infection (Figure 5b). We collected brain tissues from the dead chicks and examined the viral titers (Table 3). The titers in the brains of six chicks infected with 10^6^ PFU of rg24a2b were 3.1 × 10^6^, 2.3 × 10^5^, 1.3 × 10^5^, 2.3 × 10^5^, 9.3 × 10^3^, and 2.0 × 10^5^ PFU/g, respectively. No infectious virus was detected in the brain of the one dead chick inoculated with rg24a2bMT (10^6^ PFU/head). The viral titer in the brain of the one chick that died after infection with 10^3^ PFU/head of rg24a2b was 2.9 × 10^5^ PFU/g. We then extracted total RNA from the brain tissues and determined the HA cleavage site sequences by Sanger sequencing using RT-PCR-amplified HA genes. As expected, all of the HA cleavage sites of the viruses found in the dead chicks infected with rg24a2b had PQRKKRKKR/GLF, which was consistent with the HA cleavage site of the viruses that showed trypsin-independent viral replication in vitro (Table 2). These results indicated that all chicks infected with rg24a2b, but not rg24a2bMT, died due to replication of virus that had acquired additional basic amino acid residues at its HA cleavage site.

## 3. Discussion

It has been suggested that the secondary structure of the RNA encoding the HA cleavage site of IAVs contributes to the conversion of LPAIV to HPAIV by facilitating the acquisition of multiple basic amino acids at the HA cleavage site, but direct evidence is lacking. We previously showed that nucleotide insertions frequently occur in the stem-loop structure containing the adenine stretch in the RNA sequence encoding the cleavage site of H5 HAs [33]. In the present study, we further show that increased nucleotide insertions create additional codons for basic amino acids, resulting in the generation of viruses with the ability to replicate in the absence of trypsin.

Although the molecular mechanism underlying the insertion of additional nucleotides into RNA molecules that form stem-loop structures containing adenine stretches has not yet been revealed, similar nucleotide insertion events associated with stem-loop structures have been reported for other viruses. For example, consecutive nucleotides are used to increase the coding capacities of virus genomes. The phosphoprotein (P) gene of the paramyxovirus encodes multiple products, a process that involves the insertion or deletion of non-templated guanosine nucleosides into mRNA at a conserved slippery site that contain runs of purine nucleotides [35,36,37,38,39]. In Ebola virus replication, transcriptional slippage, where non-templated adenine residues are incorporated into glycoprotein (GP) mRNA at a slippery site containing seven adenine residues, is used to primarily produce secretory GP, and edited mRNAs are translated to membrane-anchored GP and another secretory GP [40,41,42]. The general consensus is that the secondary structure of the genome is involved in the mechanism of polymerase slippage at slippery sites containing consecutive nucleotides to produce multiple products, but the underlying mechanisms remain controversial [43,44]. In this study, the positive sense sequences (i.e., cRNA sequences) were used to predict the structure, but when negative sense sequences (i.e., vRNA sequences) were used for the prediction, the loop size was similar among Shimane, 24a, and 24a2b (data not shown). It is unclear which RNA sense is the preferable template for the insertion of nucleotides. Furthermore, since influenza virus genomic RNAs are in general protected by viral nucleoproteins immediately after being synthesized and long naked viral RNA molecules are unlikely to be exposed during the RNA replication/transcription, further studies are needed to clarify how the stem-loop structure facilitates nucleotide insertions.

HPAIVs in nature have been documented for only H5 and H7 subtype viruses, with a few exceptions, but there might be a possibility that other subtypes of LPAIVs also have the potential to evolve into HPAIVs. Indeed, stem-loop structures have been found in other IAV subtypes and in influenza B and C viruses [25,33]. Our previous comprehensive analysis of database sequences of LPAIVs isolated from ducks showed that the stem-loop structure is present in most viruses regardless of the HA subtype but varies greatly in size and position, and that most H5 and H7 subtypes contain a large loop structure consisting of eight or more nucleotides forming the codons for the cleavage site [33]. Such long and well-positioned loop structures have been found in the H4, H6, H9, H10, and H16 subtypes, but at much lower frequencies than those in the H5 and H7 subtypes [24,25,33]. H9N2 viruses with a tribasic cleavage site are found at a high frequency in Asia and the Middle East, and the RNA region encoding the HA cleavage site of these viruses is predicted to form a stem-loop structure that might be related to nucleotide insertions observed in the reporter assay [45]. While the present study supports the notion that the RNA region encoding the HA cleavage site of the H5 subtype virus contributes to the acquisition of the polybasic HA cleavage site, further study is needed to clarify whether IAVs other than those of the H5 and H7 subtypes naturally acquire a polybasic cleavage motif to develop a phenotype highly pathogenic to avian species.

In this study, additional basic amino acid insertions into the HA cleavage site (from RKKR/G to RKKRKKR/G or RRKKR/G) occurred during viral replication in MDCK cells. Although the sequence RKKKR/G showed the highest frequency in deep sequencing analysis (Table 1), viruses having this sequence were not found in the plaque assay (Table 2), suggesting that viruses with the sequence RKKKR/G might have some disadvantage in replication ability compared to those with RKKRKKR/G. The RKKRKKR/G sequence at the HA cleavage site became dominant when the virus replicated in chicks. A previous study showed that in chickens there is relatively little selection pressure on monobasic and extended (5 or more amino acid residues) HA cleavage sequences; however, mid-length (3 or 4 amino acid residues) HA cleavage sequences are rapidly replaced by extended forms [44]. Thus, it is conceivable that the viruses with a relatively short cleavage site (RKKR/G) acquired further basic amino acid residues during replication in chicks due to their enlarged loop, which may have triggered nucleotide insertions into the viral RNA.

In summary, here we investigated the biological significance of the stem-loop structure of the RNA sequence encoding the HA cleavage site. Taken together, the present data and our previous study [33] demonstrate that the presence of a stem-loop structure and an adenine (i.e., uracil in negative-sense viral RNA) stretch in the RNA region encoding the HA cleavage site is a key genetic determinant for the emergence of HPAIVs of the H5 subtype in nature. These findings provide important insights for predicting the potential of LPAIVs circulating in poultry to become HPAIVs.

## 4. Materials and Methods

### 4.1. Cells and Viruses

Madin–Darby canine kidney (MDCK) cells were maintained in Eagle’s minimal essential medium (MEM) (Sigma) supplemented with 10% calf serum (CS) (Gibco) and Penicillin-Streptomycin (100 U/mL penicillin and 0.1 mg/mL streptomycin) (Gibco). Human embryonic kidney 293T cells were grown in Dulbecco’s modified Eagle’s medium (Sigma) containing 10% fetal calf serum (Sigma) and antibiotics as described above. Quail tumor (QT) 6 cells were maintained in Kaighn’s modification of Ham’s F-12 medium (Gibco) supplemented with 5% CS, 10% tryptose phosphate broth (Difco), and antibiotics as described above. All cells were incubated at 37 °C under 5% CO₂. AIV strain A/whistling swan/Shimane/499/1983 (H5N3) (Shimane) was kindly provided by Dr. Toshihiro Ito (Tottori University), propagated in the allantoic cavities of 10-day-old embryonated chicken eggs at 35 °C for 48 h, and stored at −80 °C until use.

### 4.2. RNA Secondary Structure Prediction

RNA secondary structures were predicted by using mfold from the UNAFold Web Server (http://www.unafold.org/, accessed on 1 August 2021) [46,47,48], RNAFold from the ViennaRNA Web Services (http://rna.tbi.univie.ac.at/, accessed on 1 August 2021) [49], and CentroidFold (http://rtools.cbrc.jp/centroidfold/, accessed on 1 August 2021) [50]. The sequence corresponding to the RNA encoding the HA cleavage site of each virus and its modified sequences were used for structural analysis. The structures predicted by these 3 methods were compared and confirmed to be similar.

### 4.3. Reporter Assay

The reporter assay was carried out as described previously [33,51]. Briefly, by using the pHW72-LUC-CKpolI plasmid containing a chicken RNA polymerase I promoter, a mouse RNA polymerase I terminator, a noncoding region (NCR) from the HA segment of the A/Puerto Rico/8/34 (H1N1) (PR8) strain, and a firefly luciferase gene, the reporter plasmids were constructed by inserting 29 or 30-nucleotide linkers corresponding to the sequences encoding the HA cleavage site of the virus between the start codon and the remaining open reading frame (ORF) of the firefly luciferase gene. The eukaryotic expression plasmid pCAGGS/MCS encoding the PR8 polymerases (PB2, PB1, and PA) and NP under the control of the chicken β-actin promoter was kindly provided by Dr. Yoshihiro Kawaoka (University of Tokyo). QT6 cells grown in 24-well plates were transfected with PB2-, PB1-, PA-, and NP-expressing pCAGGS (or empty pCAGGS/MCS instead of pCAGGS/PB2) (150, 150, 150, and 300 ng, respectively), the pRL-TK Renilla luciferase transfection control reporter plasmid (Promega, Madison, WI, USA) (5 ng), and the reporter plasmids (pHW72-LUC-CKpolI plasmids) (150 ng) by using FuGENE HD (Promega). The luciferase activity in the plasmid-transfected QT6 cells was quantified by using the Dual-Luciferase Reporter Assay System (Promega) at 24 h after transfection (firefly luciferase activities were divided by Renilla luciferase activities).

### 4.4. Generation of Recombinant Viruses by Reverse Genetics

The viral RNA (vRNA) of Shimane was extracted from allantoic fluids containing virus particles by using a QIAamp viral RNA minikit (Qiagen, Hilden, Germany), and cDNA was synthesized with superscript III reverse transcriptase (Invitrogen) using the Uni12 primer (5′-AGCAAAAGCAGG). The seven gene segments (PB2, PB1, PA, NP NA, M, and NA) of Shimane were amplified and cloned into the pHH21 plasmid, which contains the human RNA polymerase I promoter and the mouse RNA polymerase I terminator separated by BsmBI sites [52]. The pHH21 plasmids encoding the HA gene of Shimane and 24a2b were reported previously [33]. The pHH21-based plasmid containing the HA gene of 24a was constructed by mutagenesis using the plasmid carrying the HA gene of Shimane. To generate plasmids containing the mutated 24a2b HA gene, the 24a2b HA gene was amplified as two fragments using primers with the required mutations and BsmBI site. The two amplified fragments were digested with BsmBI (New England Biolabs) and ligated into the pHH21 plasmid. The pHH21-based plasmid for the expression of the PB2, PB1, PA, NP, NA, M, and NS of PR8 was kindly provided by Dr. Yoshihiro Kawaoka (University of Tokyo). Recombinant viruses (rgPR8/Shimane, rgPR8/24a2b, rgPR8/24a2bMT, rgShimane, rg24a, rg24a2b, and rg24a2bMT) were generated by using a reverse-genetics system as described previously [52] with slight modification. Briefly, 293T cells were transfected with 8 pHH21-based plasmids providing viral RNAs of each segment (500 ng) and the pCAGGS-based plasmids expressing PB2, PB1, PA, and NP of PR8 (1 μg) using TransIT-LT1 (Mirus Bio, Madison, WI, USA) according to the manufacturer’s protocol. Supernatants were collected at 48 h post-transfection and the viruses were propagated in MDCK cells and stored at −80 °C until use.

### 4.5. Plaque Assay

Confluent MDCK cells in 6-well plates were infected with 10-fold dilutions of each virus or tissue homogenate. After a 1-h incubation, the inoculum was removed and the cells were overlaid with 1 × MEM (Gibco, Waltham, Massachusetts, USA) containing 0.3% bovine serum albumin (Sigma, St. Louis, MO, USA), 1 × MEM Amino Acids Solution (Gibco), 1 × MEM Vitamin Solution (Gibco), 2 mM L-Glutamine (Gibco), 0.3% Sodium Bicarbonate (Gibco), Penicillin-Streptomycin (100 U/mL penicillin and 0.1 mg/mL streptomycin), and 1% Bacto agar (Becton Dickinson, Sparks, MD, USA) and were incubated in the presence or absence of 5 μg/mL trypsin at 37 °C. Then, the cells were stained with 0.25% crystal violet solution containing 10% formaldehyde. Plaques were counted and viral titers were determined as plaque forming units (PFU). Experiments with HPAIVs were carried out in the biosafety level 3 (BSL-3) facility at the International Institute for Zoonosis Control, Hokkaido University, Japan.

### 4.6. Sequence Analysis of the Hemagglutinin Genes of Plaque-Cloned Viruses

Viruses were collected from randomly selected plaques (n = 9) by using sterile filter tips and suspended in MEM. RNA was extracted from the suspensions by using a QIAamp viral RNA Mini Kit (Qiagen) (extracted without carrier RNA). Reverse transcriptase-polymerase chain reaction (RT-PCR) amplification was performed using a PrimeScript High Fidelity RT-PCR Kit (TaKaRa, Kusatsu, Japan) with a primer pair that flanked the cleavage site region. Nucleotide sequences were determined by Sanger sequencing.

### 4.7. Deep Sequencing

Deep sequencing analysis was performed as previously reported [33] with some modifications. MDCK cells were infected with rgPR8/Shimane, rgPR8/24a2b, or rgPR8/24a2bMT at a multiplicity of infection (MOI) of 0.001 and incubated at 37 °C for 48 h. Then, virus particles in the supernatant were concentrated by high-speed centrifugation and purified through 20–40% sucrose gradient ultracentrifugation. RNA from the purified virus particles was extracted by using TRIzol LS reagent (Sigma) according to the manufacturer’s protocol. cDNA libraries were prepared from vRNA without any amplification procedures and sequenced to generate high depth of coverage (more than 100,000 reads) with a high-quality score (not less than 30) as follows: double-stranded cDNA of the partial HA gene containing the sequence encoding the HA cleavage site was synthesized using the PrimeScript double-strand cDNA synthesis kit (TaKaRa) with the HA gene-specific primer H5-963F (5′-GTATGCCTTTCCACAATATTCATCC). cDNA libraries tagged with sequencing adapters with indexes specific to each sample were obtained by using a TruSeq DNA PCR-free sample prep kit (Illumina, San Diego, CA, USA). The quality of the libraries was checked on a Bioanalyzer (Agilent Technologies, Santa Clara, CA, USA) using a High Sensitivity DNA chip. The libraries were quantified with real-time PCR using the KAPA Library Quantification Kit (KAPA Biosystems, Wilmington, MA, USA). The prepared libraries were sequenced on a MiSeq platform using a 600 cycle V3 kit (Illumina) to perform 300-bp paired-end sequencing, following the manufacturer’s protocol. Sequenced reads were divided by barcodes. Illumina Miseq reads were mapped to the reference HA sequence from Shimane determined by Sanger sequencing using bowtie 2 [53] with default parameters. Alignments corresponding to the 29-nucleotide linker sequence were examined to count the frequency of derived insertions using scripts developed in-house. Reads with quality scores lower than 30 were eliminated.

### 4.8. Animal Experiments

All animal experiments were conducted in strict accordance with the Guidelines for Proper Conduct of Animal Experiments of the Science Council of Japan. The protocol was approved by the Animal Care and Use Committee of Hokkaido University on March 30, 2018 (#18-0026). Zero- to 3-day-old chicks (6 for each group) were intracerebrally inoculated with 100 μL of each virus (10^6^ PFU/head or 10^3^ PFU/head) and monitored for 10 days. Brain tissues from the chicks that died (or were euthanized at a human endpoint) during the monitoring period were collected. To check the viral titers, the brain samples were homogenized in transport medium consisting of MEM containing 0.5% bovine serum albumin fraction V (Roche, Basel, Switzerland), 10,000 U/mL penicillin G (Meiji Seika, Tokyo, Japan), 10 mg/mL streptomycin (Meiji Seika), 0.3 mg/mL gentamicin (Nichi-Iko, Toyama, Japan), and 250 U/mL nystatin (Sigma) and then centrifuged. The supernatant (10% homogenate) was used for plaque assays. To determine the amino acid sequence of the cleavage site, total RNA was extracted from the brain samples by using TRIzol reagent (Sigma) and RT-PCR was conducted as described. These experiments were carried out in the BSL-3 facility at the International Institute for Zoonosis Control, Hokkaido University, Japan.

## Figures and Tables

**Table 1:** Amino acid insertions at the HA cleavage sites encoded by vRNA in rgPR8/24a2b particles.

Amino Acid Sequence ^a^	Number of Reads (*n* = 12,168)	Percentage of Total Reads (*n* = 4,248,638)
QRKKKRGLFG	5415	0.127
QRKKRKKRGLFG	1946	0.046
QREEKRGLFG	1897	0.045
QRRKKRGLFG	1749	0.041
QRKKKKRGLFG	742	0.017
Others ^b^	419	0.010

^a^ Amino acid sequences of the top five read numbers are shown. ^b^ Sequences from other reads including nucleotide insertions associated with in-frame codons.

**Table 2:** Sequences of HA cleavage sites of viral clones grown in the presence or absence of trypsin.

Virus	Trypsin	Nucleotide Sequence Amino Acid Sequence									
rg24a2b	+	AGA	- - - ^a^	- - -	- - -	AAA	AAA	AGA	GGT	CTG	TTT
		R	-	-	-	K	K	R	G	L	F
	−	AGA	AAA	AAA	AGA	AAA	AAA	AGA	GGT	CTG	TTT
		R	K	K	R	K	K	R	G	L	F
		AGA	- - -	- - -	AGA	AAA	AAA	AGA	GGT	CTG	TTT
		R	-	-	R	K	K	R	G	L	F
rg24a2bMT	+	CGC	- - -	- - -	- - -	AAG	AAG	AGA	GGT	CTG	TTT
		R	-	-	-	K	K	R	G	L	F
	− ^b^										

^a^ Dashes are included to adjust for sequence alignment. ^b^ No visible plaque was found.

**Table 3:** Titers and cleavage site sequences of viruses isolated from the brains of chicks inoculated with rg24a2b or rg24a2bMT.

Inoculum	Virus	Titer (PFU/g) ^a^	Nucleotide Sequence Amino Acid Sequence									
10^6^ PFU/head	rg24a2b	3.1 × 10^6^	AGA	AAA	AAA	AGA	AAA	AAA	AGA	GGT	CTG	TTT
			R	K	K	R	K	K	R	G	L	F
		2.3 × 10^5^	AGA	AAA	AAA	AGA	AAA	AAA	AGA	GGT	CTG	TTT
			R	K	K	R	K	K	R	G	L	F
		1.3 × 10^5^	AGA	AAA	AAA	AGA	AAA	AAA	AGA	GGT	CTG	TTT
			R	K	K	R	K	K	R	G	L	F
		2.3 × 10^5^	AGA	AAA	AAA	AGA	AAA	AAA	AGA	GGT	CTG	TTT
			R	K	K	R	K	K	R	G	L	F
		9.3 × 10^3^	AGA	AAA	AAA	AGA	AAA	AAA	AGA	GGT	CTG	TTT
			R	K	K	R	K	K	R	G	L	F
		2.0 × 10^5^	AGA	AAA	AAA	AGA	AAA	AAA	AGA	GGT	CTG	TTT
			R	K	K	R	K	K	R	G	L	F
10^6^ PFU/head	rg24a2bMT	ND ^b^	CGC	- - - ^c^	- - -	- - -	AAG	AAG	AGA	GGT	CTG	TTT
			R	-	-	-	K	K	R	G	L	F
10^3^ PFU/head	rg24a2b	2.9 × 10^5^	AGA	AAA	AAA	AGA	AAA	AAA	AGA	GGT	CTG	TTT
			R	K	K	R	K	K	R	G	L	F

^a^ Virus titers in the brain tissues were determined by use of plaque assays with MDCK cells. ^b^ Infectious virus was not detected but the HA gene (RKKRGLF) was detected in the brain homogenate. ^c^ Dashes are included to adjust for sequence alignment.

## Data Availability

The data presented in this study are available on request from the corresponding author.
